# Mental health & resilience in crews of emergency medical service helicopters - load analysis of stressful missions

**DOI:** 10.3389/fpsyt.2026.1768489

**Published:** 2026-03-03

**Authors:** Sabrina Ziehr, Florian Kramer, Krystian Pracz

**Affiliations:** DRF Stiftung Luftrettung gGmbH, Filderstadt, Germany

**Keywords:** burdensome operations, HEMS (helicopter emergency medical service), mental health, peer-support, resilience

## Abstract

Crews of rescue transport helicopters (RTH) are repeatedly confronted with difficult missions and operational scenarios. When dealing with and processing difficult missions in particular, PSNV services (PSNV – psychosocial emergency care) are rarely taken up. Even actively offered assistance is usually declined, regardless of who makes the offer. To investigate the number of potential stressful missions, e.g. pediatric patients with NACA-score 5-7, suicide or suicide attempts, second victim aspect or critical incidents, and the use of PSNV at the DRF Luftrettung were analzyed using systematically generated data over a two-year-period. 1383 missions were extracted from the data. Overall, 4 contacts of reaching out for help and support could be identified. Corresponding to the literature, the data suggests that crews do not feel the need to be supported to overcome difficult missions despite the debriefing in the crew. It leads to the assumption that the feeling of safety, respect and teamwork provides a great resource in dealing with burdensome missions. But it must also be discussed that maybe there exists a number of unrecorded cases. For that, further research in qualitative design of resources and challenges for the resilience and mental health of the crews of emergency medical service helicopters is needed to get better understanding of the phenomenon and support the emergency-personnel’s well-being and human performance.

## Introduction

Air rescue personnel (pilot, HEMS-TC, emergency physician) are exposed to high levels of stress due to the special requirements of their missions (1). This is reflected in medical personnel, for example, in increased stress on flight days compared to days off or days with ground-based service (2). Stressors in air rescue can be divided into hazard-related and non-hazard-related stressors (3). Thereby factors relating to the treatment of patients are not considered. A distinction must furthermore be made between external and psychosocial stress factors (1). Special external factors include temperature, noise levels, movement, and weather conditions during flight (4). This requires a certain level of physical fitness and health (5). These challenges are usually taken on by people who are characterized by composure, calmness, and emotional stability. In addition, they work conscientiously, as even the smallest mistakes can have a massive impact on the outcome of the mission (6). On a psychosocial level, pressure to perform and work-related overload are the biggest stressors in challenging rescue missions (1, 5). Petrowski and colleagues (7) were able to establish that pediatric missions and time-critical diagnoses, such as stroke or cardiovascular events, cause the highest levels of stress.

Pietsch and colleagues (8) evaluated Swiss mission data and found that 20% of missions involved serious health-threatening diagnoses. Furthermore, the first missions on duty cause the most hormonally induced stress (7). In addition, the work interacts with the private environment, which manifests itself in social overload and isolation as well as chronic worries (2).

In principle, there appears to be an increased risk of developing PTSD or other mental health problems (1). This contrasts with the findings of Reid and colleagues (9), who discovered in their study that Norwegian air rescue personnel do not exhibit an increased incidence of PTSD, anxiety, or depression. They conclude that the personnel can be classified as resilient. They cite training, education and experience as key markers for the development of resilience. Other resources for reducing stress include personal character and one’s own sense of coherence. The higher the subjective well-being, the greater the self-efficacy and self-confidence, which reduces chronic stress (1). In addition, the work environment is of central importance, which on the one hand is psychosocially characterized by support (1) and on the other hand leads to greater self-confidence and satisfaction through specific training such as CRM, and consequently also to increased patient safety (10). Especially given the high proportion of diagnoses of serious health threads, it is clear that air rescue personnel must be experienced and specially trained (8). Training and education, as well as robust risk analyses, strengthen personal safety, which includes, above all, debriefings after missions and structured follow-up discussions (3). Not to be neglected as well are physical fitness and health, which can help reduce incidents and accidents, leading to a positive outcome both personally and for the company in terms of the operational readiness of air rescue personnel (11).

In their study on resilience in the healthcare sector with emergency responders, Thielmann, Ifferth & Böckelmann (12) found that people with higher resilience experience less stress, as measured by lower levels of irritation and burnout symptoms, than people with lower levels of resilience. Higher performance at work was also derived for the more resilient group. Higher resilience also has a positive effect on flight safety in air rescue. Among other things, increasing disruptive factors (e.g., extreme weather, open landing sites), constant complex human-machine interaction, and 3 instead of 2 dimensions further increase the number of stressors. If resilient individuals experience less stress, it can be assumed that they also experience less irritation and therefore make fewer mistakes. Both of these factors have a direct impact on flight safety during operations as well as during preparation and follow-up.

DRF Luftrettung, one of Europe’s most experienced and largest air rescue organizations. They operate in four countries (GER, AUT, LIE, CH) in different environments. Based on those differences in the environment (mountains, flatlands, coastline and offshore), the crew composition varies alongside with the environment and the corresponding mission profile. The typical crew is composed by a pilot, a HEMS-TC, an emergency physician. During the night the flightcrew is extended by an additional pilot. In the mountain area or in case the helicopter is equipped with a hoist, additional personal is required (helicopter hoist operator - HHO, mountain rescuer). For all crew members a long professional track record is required (flight hours, years of experience as paramedic) including specific educational elements, before entering the helicopter emergency service. In addition, DRF is also engaged in the flied of disaster control and civil-military cooperation. As a response to all those different challenges, DRF Luftrettung is offering a range of preventive measures. These include the Flight Crew Support Program (13) and regular CRM training for crews, that also focus on topics such as pressure, cooperation, and resilience. In addition, a specific resilience project within the company addresses the topics of health, well-being, resilience, and other aspects that promote resilience. Stressful mission situations are recorded in a First Aid Logbook. Those who make an entry then receive an email with an individual support offer, which they can make use of, but are not obliged to. In addition, crew members have the option of contacting an external professional peer hotline 24/7 on their own initiative.

**Figure 1:**
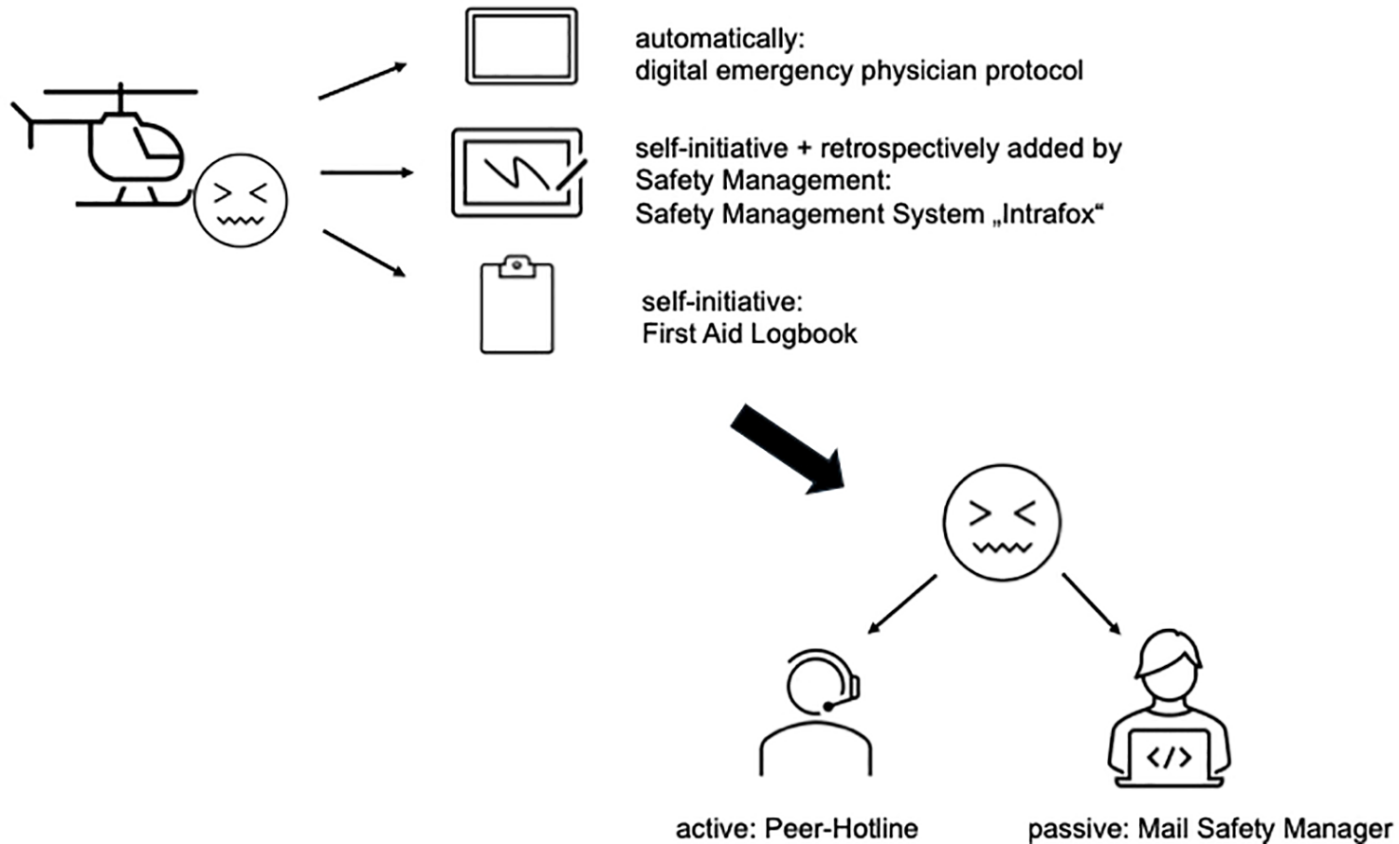
Registration of potentially stressful missions.

**Figure 1** schematically illustrates how potentially stressful missions within DRF Luftrettung can be registered and what options are available for obtaining support.

As shown in the literature crews of rescue transport helicopters are often confronted with stressful events at work (8). They can be divided in hazardous and non-harzardous stressors (3). Most stressful situations are for example pediatric emergencies or time-critical diagnoses (7). The impact of these missions on the crews remains unclear. Whereas there are references, that a higher risk of PTBS exists (1), there are clues in contrast, too (9). The latter can be related to the findings that the persons working as flight rescuer have character traits which lead to more resilience (9). Based on the literature the first aim of thy study is to assess the status quo of potentially stressful operational situations at the DRF-Luftrettung. The second aim is to investigate how often supporting systems are taken up. To answer the question for the crews within the DRF Luftrettung a two-year-period of missions is investigated.

## Methods

To analyze the status quo, various data sets on stressful mission situations were retrospectively evaluated in a quantitative-descriptive manner for the observation period August 1, 2022 – September 1, 2024.

### Material & procedures

The following available data sets were evaluated by the safety manager of the DRF-Luftrettung:

- Digital emergency physician protocol to determine the type of mission
- Safety Reporting System (Intrafox) for safety-related near miss events and incidents
- Entries in the First Aid Logbook
- Use of support

The total count of primary rescue missions at the DRF-Luftrettung during the two-year-period was 52266. Due to the interesting events 1383 of them were included in the analysis. The included data was filtered out of the emergency physician protocols by using NACA Score and the entries of event risks in the safety management reporting system.

The NACA score is an established scoring system in emergency medicine in Germany and is used to categorize the severity of injuries, diseases or poisonings (14). Scoring is made by the emergency physician. Consistent to the literature, that especially time-critical diagnoses have a great impact on stress (7), NACA Score V – VII were included. These involve:

- NACA V – acute danger
- NACA VI - respiratory and/or cardiac arrest
- NACA VII – death

Using these scores pediatric emergencies, violent crimes, suicide or attempted suicide can be included in the sample.

**Figure 2:**
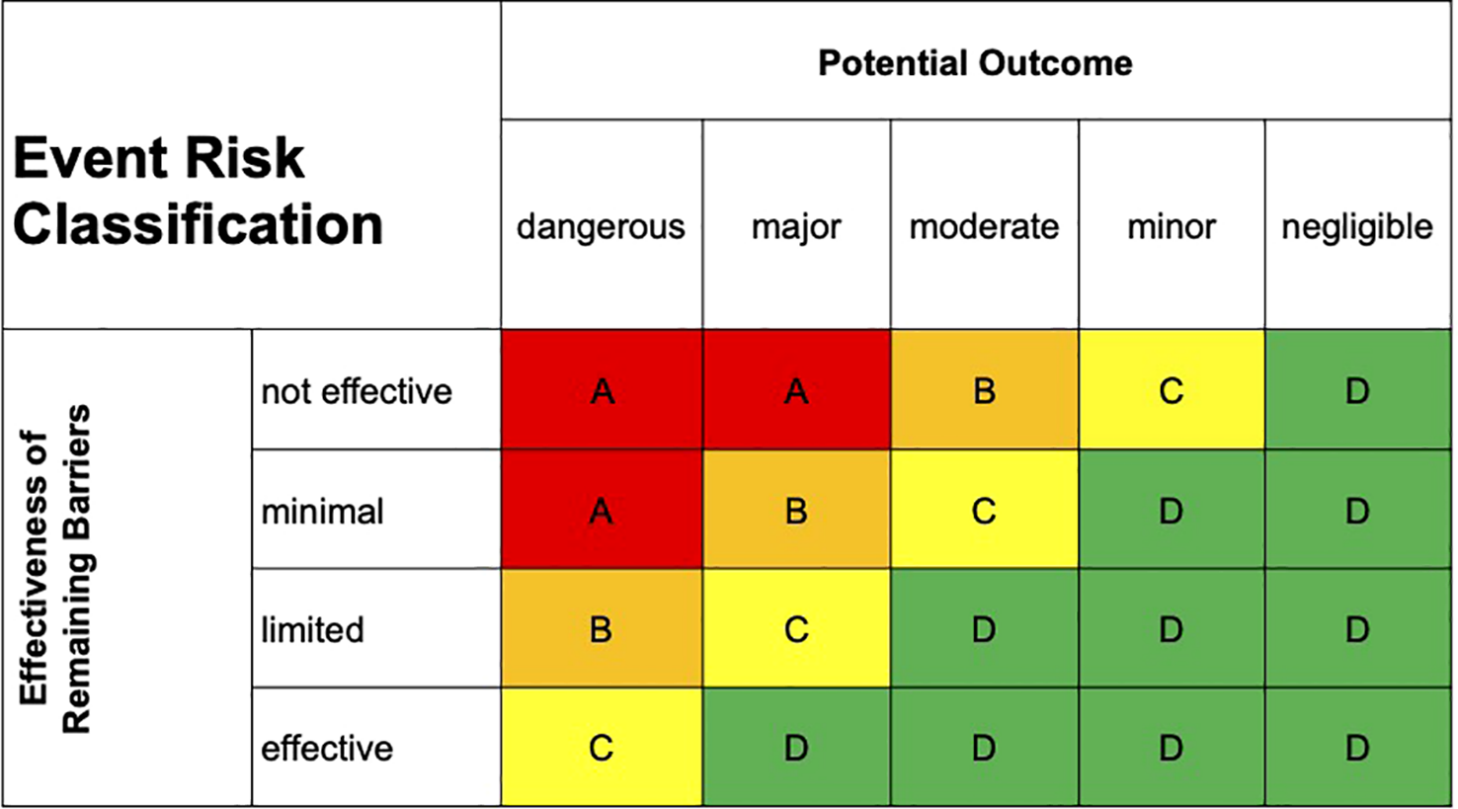
Modified ERC rating used at DRF Luftrettung.

To filter the missions in the context of risk the Intrafox Safety Reporting System was used. At DRF a modified version of the established ERC rating (15) is used for. In addition to the existing 3 dimensions red, yellow and green, orange is established for critical incidents. **Figure 2** shows the classification system.

As dangerous are classified as total loss of an aircraft, fatal injury to persons or very large third-party damage. Major effects are described as severe damage to the aircraft, serious injury to persons or major third-party damage. Moderate incidents include significant damage to the aircraft, minor injury to persons (medical intervention required) or moderate third-party damage, whereas minor events have minor impact on the aircraft, the persons in rescue or third party damage. If the outcome had no damage to the aircraft or persons or third parties, the events were classified as negligible.

In total there were 871 reports during the observation period. Only entries classified as criticality levels A and B-Events were included which leads to 46 relevant events. The variability of these events is high. It includes undesirable events in emergency medicine and flight operations, such as air proximities or (nearby-)collisions with ground obstacles.

The anonymous entries in the First Aid Logbook were manually included in the evaluation based on the free text information provided in the description of the incident regarding perceived psychological stress. Due to the small sample size and double recording, e.g., also via the digital emergency physician protocols, results by the First Aid Logbook were not included separately in the evaluation. To investigate the extent and way support is utilized after potentially stressful operations, the number of initial contacts to the PSNV hotline was evaluated.

All data is collected anonymously so that no conclusions can be drawn about the person entering the data.

In retrospect, it cannot be determined whether the entries in the different media refer to the same critical events.

### Analysis

The available data sets were evaluated using explorative quantitative descriptive statistics using sum. To operationalize potentially stressful incidents, hazard-related and non-hazard-related stressors were used in accordance with existing literature (3). Hazard-related stressors include: threats to personnel, perceived danger to life, errors with major consequences, and disaster situations. Personal events that are not necessarily hazard-related include: serious pediatric emergencies, suicides or suicide attempts, violent crimes, and incidents in the personal environment.

## Results

The results are presented according to the classification of hazard-related and non-hazard-related stressors (3). In addition, data on the use of support after potentially stressful operations are presented. Where possible, the different data sources are correlated with each other. **Table 1** gives an overview of identified missions.

**Table 1:** Hazard-related and non-hazard-related stressors within the observation period.

Category	Characteristic	Number	Datapool
Hazard-related Stressors	Aggression to personnel	2	Safety Reporting System
	Critical events	38	Safety Reporting System
	Human errors with major consequences	6	Safety Reporting System
Non-hazard-related Stressors	Serious pediatric emergencies	1048	Digital emergency physician protocols
	Suicides or suicide attempts	216	Digital emergency physician protocols
	Violent crimes	73	Digital emergency physician protocols

### Hazard-related stressors

The available data provided information about 46 hazard-related stressors such as threats to personnel, perceived danger to life, and errors with major consequences. Aggression to personnel were identified two times (4%) using the safety reporting system. In these cases, pilots were threatened with a knife during the mission. Critical incidents and near misses were recorded a total of 38 times (83%). In addition, there were 6 (13%) classified as an individual error according to the Human Factor Classification System (HAFCS; 16). In connection with the potential outcome, there is a risk of a second victim phenomenon. There were no reports of disaster situations during the period analyzed.

### Non-hazard-related stressors

**Figure 3:**
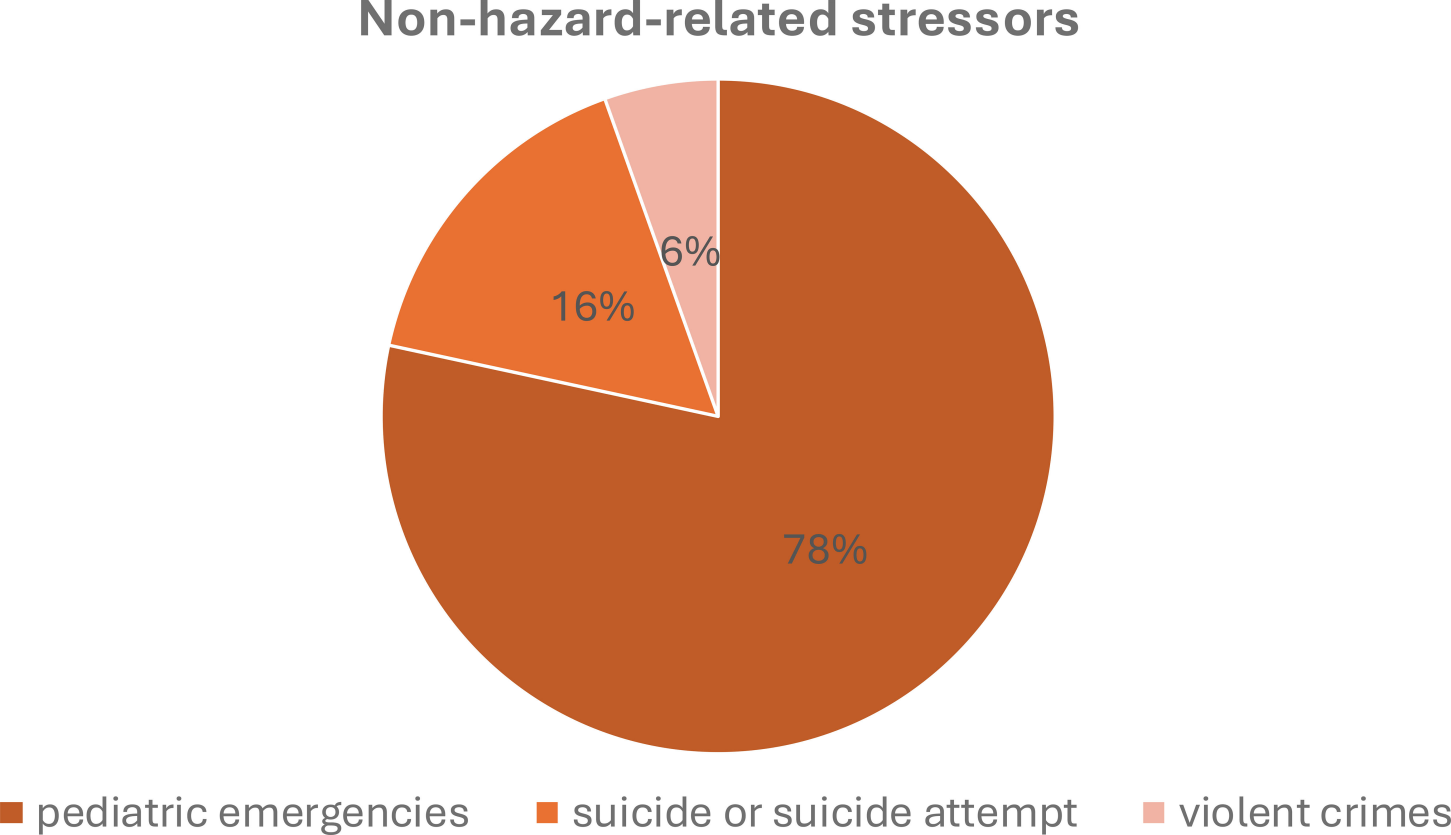
Non-hazard-related stressors.

From the non-hazard-related stressors derived from the literature (e.g. 7), data on missions relating to pediatric emergencies, suicides, and violent crimes could be extracted. A total of 1337 missions in this category were identified during the observation period. These were recorded in full via the digital emergency physician protocols. As shown in **Figure 3**, 78% of missions 78% of missions were pediatric emergencies, 16% were incidents related to suicide or suicide attempt and 6% were violent crimes.

In total, crews were called to 1048 pediatric emergencies (ages up to 16) with acute life-threatening conditions, resuscitation, or death (NACA 5-7) during the observation period. Among these, 88% (n=920) of missions dealt with acute life-threatening situations, while 11% (n=111) were related to resuscitation efforts. A total of 2% (n=17) deaths occurred at the scene.

There were 216 incidents related to suicides or suicide attempts with a NACA 5-7, of which a total of 46 (21%) resulted in the death of the patient. Violent crimes were recorded a total of 73 times.

### Use of support options

During the observation period, a total of four contacts with the PSNV hotline were identified, with both email and telephone contacts being counted as initial contacts by the individuals themselves. In addition, the safety manager contacted those who had entered information in the First Aid Logbook to offer support or follow-up, but these offers were not taken up.

## Discussion

Data analysis shows that DRF Luftrettung air rescue personnel were exposed to a total of 1383 potentially stressful mission situations during the observation period. These can be divided into hazard-related and non-hazard-related events (3). Hazard-related events are primarily reflected in errors with major consequences at Level A or B according to ERC (15) (n=6) as well as 38 other critical and near-miss events or two missions with aggression to personnel. Errors with major consequences are worth considering for further analysis, as their classification as errors may lead to feelings of guilt on the part of the persons involved. In terms of appropriate error and risk management, recording and dealing with these errors and incidents is essential to strengthen the perceived technical safety of air rescue personnel. Apart from this technical aspect, however, perceived safety at the interpersonal level also plays an important role (1, 10). The data from the observation period shows that the crews themselves can also become victims, for example, if they are exposed to aggressive attacks during the mission. Even though the total number of two in the context of these entries seems relatively low, it is important to discuss the fact that these entries were made in the safety management system. The sensitivity of this issue can be derived, in part, from the fact that the data must be entered there on the individual’s own initiative and not by the system. Research has shown that aggressive attacks become more often and have a negative impact on the rescue personnel (17).

The data collected on non-hazard-related stressors (3) is consistent with the existing literature (7, 8). Most of the incidents in this category are related to pediatric emergencies. 1048 data entries were derived from the digital emergency physician protocols. This is also consistent with the study results of Petrowski and colleagues (7), who were able to show that such missions are perceived as highly stressful and burdensome. Missions in the private sphere are also worthy of special attention, even though they were not recorded via the Safety Management System or digital emergency physician protocols.

Despite the high frequency of potentially stressful events (n=1383), offers of support were only used extremely rarely, namely under 1% (n=4). As in the literature, this result may be related to the fact that people who work in this field tend to be stable and resilient (5). Furthermore, a significant positive factor may be the sense of coherence and cohesion with the actual mission, namely saving lives (1). In the latest employee survey conducted by DRF Luftrettung, the meaningful and motivating work in air rescue was highlighted as a particularly positive feature of the workplace. Teamwork and cohesion were also highlighted as positive examples. Here, targeted measures such as regular CRM training, further joint team-building activities, and close communication with supervisors can contribute to a healthy working atmosphere and thus to the prevention of stress. These factors are also mentioned in the literature as preventive aspects (1, 10). In addition, informal conversations with the crews at the DRF Luftrettung air rescue bases reveal a good team dynamic within the crews, but also at the bases themselves. Finally, the morning briefing, which is carried out independently, as well as the debriefing after a mission, can counteract increased stress (3). The corporate culture, which is significantly influenced by the chosen Just Culture approach, should also be considered important. In addition to meaningful and motivating work, trust is another fundamental aspect. The aforementioned CRM training, fatigue management, communication culture, as well as the right experience and decision-making skills are all elements that shape the culture and have a positive impact on the environment at work (1). However, trust is the basis for the corporate culture and is particularly influenced by “just culture” – with a corresponding effect on resilience.

In conclusion, it can be stated that, despite the high frequency of potentially stressful missions during the observation period, the crews of DRF Luftrettung make little or no use of support services. It is worth discussing at this point whether there is a significantly higher number of unreported cases resulting from the fact that potentially affected individuals neither enter information into the First Aid Logbook or the Safety Management System on their own initiative, nor seek contact with the Flight Crew Support Program (13) or the peer hotline. This would be consistent with the findings of Mikutta and colleagues (1), who identified an increased risk of developing PTSD. It is also conceivable that the crews belong to a more resilient group (9, 12), which can be considered calm, composed, and emotionally stable (5). However, this contrasts with older data documented by DRF Luftrettung (covering the period up to 2022), which provides detailed information on the personal experiences of such missions.

It shows that crisis interventions were activated between 199 and 658 times for relatives/those involved. Consistent with the data from the observation period, follow-up care was not requested by the crews involved. The central coping element also here appears to be debriefing within the crew, which was reported in between 15 and 26 cases. For practical implications, it can be concluded that a more detailed examination of the subjective experience should be carried out with specific questions about the perceived stress.

The central limitation of the study is its explorative character and the missing detailed data. The presented study gives an overview about potential stressful missions in the DRF Luftrettung during a two-year-period. Because of the lacking specific information of perceived stress of the crews a clear connection to the stressful events is not possible. The idea of unreported cases may be one potential explanation but also the higher resilience of the personnel as reported in the literature (5, 9) or the positive atmosphere at work (1) can be relevant. Further research should take a closer focus on the resilience of the crews to strengthen potential and resources and to specifically identify risks of mental stress, including burnout and PTSD, aiming to maintain health and performance. In this context, it seems advisable to bring the focus of resilience to the forefront of research, to identify protective factors, and to correlate them with the high levels of stress described in the literature to date. Qualitative research seems to be appropriate to get more detailed information (18, 19). By reconstructing the subjective perspective of the target group, it is possible to investigate in detail protective factors as well as limiting factors in the context of peer support or PSNV. For example, qualitative interviews can get a deeper dive into challenges and resources and can highlight necessary modification in managing stressful events at work. In addition to the data collected systematically, these subjective experiences and observations must also be included in the data collection, evaluation, and interpretation, and a focused determination of a degree of resilience should be pursued. This will inevitably contribute to increased flight safety. That is the approach DRF Luftrettung is taking by launching its own resilience project that looks at system and corporate resilience in different areas of the company. At the same time, the documentation has been extended to include a First Aid Logbook so that subjective measures can also be recorded during stressful missions. Nevertheless, the implementation of systematic recording and target group-oriented debriefing remains a broad field that contributes to the effective debriefing of critical mission situations to ensure greater safety in the workplace.

## Data Availability

The data analyzed in this study is subject to the following licenses/restrictions: This is internal company data that is regularly collected as part of safety management. Requests to access these datasets should be directed to Florian Kramer (florian.kramer@drf-luftrettung.de).
